# A retrospective analysis of clinical outcome of patients with chemo-refractory metastatic breast cancer treated in a single institution phase I unit

**DOI:** 10.1038/sj.bjc.6605812

**Published:** 2010-07-27

**Authors:** A T Brunetto, D Sarker, D Papadatos-Pastos, R Fehrmann, S B Kaye, S Johnston, M Allen, J S De Bono, C Swanton

**Affiliations:** 1 The Royal Marsden Hospital Drug Development and Breast Units and Institute of Cancer Research, Sutton, UK; 2 Translational Cancer Therapeutics Laboratory, Cancer Research UK, London Research Institute, UK

**Keywords:** metastatic breast cancer, phase I trials, drug resistance

## Abstract

**Background and methods:**

Novel approaches to treat chemo-refractory metastatic breast cancer (MBC) are currently under investigation. This retrospective series reviews the outcome of 70 MBC patients who have participated in 30 phase I trials at the Royal Marsden Hospital from 2002 to 2009.

**Results:**

The median treatment lines before phase I trial entry for MBC was 5 (range: 1–12 lines). The overall response rate was 11.4% (95% CI: 4.0–18.9%) and the clinical benefit rate at 4 months was 20% (95% CI: 10.6–29.3). The median time to progression was 7.0 weeks (95% CI: 6.4–7.5) and median overall survival was 8.7 months (95% CI: 7.6–9.8) from start of first phase I treatment. No patients discontinued trial because of treatment-related toxicities. Abnormal lactate dehydrogenase, serum albumin <35 mg  per 100 ml, ⩾5 previous treatment lines, liver metastases and Eastern Cooperative Group performance status ⩾2 at study entry were significantly associated with poor overall survival in multivariate analysis.

**Conclusion:**

This retrospective analysis provides evidence that patients with MBC tolerate phase I clinical trials and a significant proportion of patients with chemo-refractory disease, particularly those with triple-negative or Her2-positive breast cancer, may benefit from treatment.

In spite of the recent advances in drug development, most women with metastatic breast cancer (MBC) have a limited median survival time of approximately 18–24 months, and only around 20% will be alive 5 years after the initial diagnosis of metastatic disease (Smigal *et al*, 2006; Chia *et al*, 2007b). The main aims of treatment are to reduce disease-related symptoms and improve quality of life and secondarily to prolong progression-free survival (PFS) and overall survival (OS) (Smith, 2006). The treatment of MBC includes chemotherapy, endocrine therapy, radiotherapy, bisphosphonates, biological therapy or combinations of these, together with supportive care and, in certain cases, resection of primary tumour or other surgical procedures. Selection of treatment is made on an individual basis taking into consideration patient- and disease-related factors (Guarneri and Conte, 2009). Although regulatory authority approval for first-line treatment is based on OS data from randomised clinical trials, approval for second- and third-line treatment is usually based on time to progression (TTP) or PFS (Cortazar *et al*, 2008).

Although MBC is usually sensitive to initial lines of standard approved drugs, acquired drug resistance and treatment failure is almost inevitable. The mechanisms of resistance are complex and can occur at many different levels. These include increased drug efflux and decreased drug influx, drug inactivation, alterations in drug target, processing of drug-induced damage, evasion of apoptosis and activation of alternative cellular survival pathways (Longley and Johnston, 2005; Gonzalez-Angulo *et al*, 2007). Novel approaches to overcome resistance are currently under investigation with new targeted agents against specific survival pathways and some patients who remain sufficiently well are offered early experimental phase I trials.

Appropriate advice at this stage of the disease course for patients with MBC remains uncertain because of the limitations of existing literature documenting patient outcome in early phase clinical trials. A recent retrospective analysis published by the MD Anderson Cancer Center (Wheler *et al*, 2010) specifically reviews the outcome of patients with MBC who have participated in phase I clinical trials in a single institution. In this study, patients have a median OS of 6.7 months; baseline characteristics of heavily pretreated disease, low albumin and poor Eastern Cooperative Group (ECOG) performance status (PS) are significantly associated with a shorter survival. Hence, we have performed an analysis in a UK institution to further characterise this cohort of breast cancer patients.

## Materials and methods

The outcome of all patients with MBC who were treated in 30 phase I trials in the Drug Development Unit at the Royal Marsden Hospital (RMH) between October 2002 and October 2009 was analysed. For those patients who had participated in more than one phase I trial, only the first trial entry was considered for survival analysis. All patients had progressive disease (PD) at study entry. Baseline characteristics were all collected within 2 weeks before starting a phase I trial. Patients were observed in weekly visits for adverse event and concomitant medication recording. Response to treatment was monitored by MRI or CT scan every 6–8 weeks and reviewed by internal consultant radiologists according to response evaluation criteria in solid tumours (Therasse *et al*, 2000). Patients continued on trial if there was evidence of response to therapy or stable disease (SD). Dose adjustments for toxicity were permitted and tumour histology was reviewed in the RMH pathology laboratory.

The hospital electronic database and clinical notes were used to collect date of diagnosis of metastatic disease, start and finish dates of phase I therapy, previous treatment lines, radiological response, date of disease progression, last follow-up and death. Variables that were included in the final univariate and multivariate analyses were: age, gender, sites of disease, treatment lines, tumour pathology, PS and laboratory parameters. The SPSS Program (Version 12.0; SPSS, Chicago, IL, USA) was used for statistical analysis. The Kaplan–Meier method was applied to estimate TTP and OS. The Cox regression model was applied for the estimation of the hazard ratio (HR); and for the multivariate analysis using a forward selection. Log-rank test (Mantel–Cox) was used to compare survival distributions. All *P*-values presented are two sided.

## Results

A total of 70 patients with MBC who participated in 30 different phase I trials at the RMH over 7 years between October 2002 and October 2009 were analysed. The start date was chosen because of the expansion in early phase trials using targeted therapeutics that commenced from this period. Baseline characteristics are shown in Table 1. The median age at the start of phase I therapy was 51.5 years (range: 34–82 years) and the median number of cycles on a phase I study was 2 (range: 1–11 cycles). The median time from diagnosis of metastatic disease to start of phase I treatment was 24 months (range: 2–102 months); the total median number of lines of treatment for metastatic disease was 5 (range: 1–12 lines) comprising 1 line of hormonal therapy (range: 0–4) and 4 of chemotherapy (range: 0–8 lines). In total, 65 out of 70 patients (92.9%) had received adjuvant chemotherapy. More than 50% of patients had received anthracycline, taxane, capecitabine and vinorelbine chemotherapy-based regimes before phase I trial entry. In all, 12 out of 70 (17.1%) patients had received previous biological therapies, excluding trastuzumab, (bevacizumab 4 patients; lapatinib 8 patients). The median number of sites of metastatic disease before phase I trial entry was 3 (range: 1–5 sites); and the most common sites were: distant lymph nodes (90%), liver (52.8%), lung (52.8%), skin–chest wall recurrence (44.2%) and bone (37.1%). In total, 8 out of 70 patients (11.4%) had an ECOG PS of 2 at study entry.

In all, 26 out of 70 (37.1%) patients had triple-negative breast cancer (TNBC), 23 out of 70 (32.9%) were oestrogen receptor (ER)-positive/HER2-negative and 21 out of 70 (30.0%) were HER2-positive. Table 2 reviews the outcome and main class of targeted agents used within these trials based on breast cancer subtype. Overall 8 out of 70 patients (11.4%, 95% CI: 4.0–18.9%) obtained a partial response (PR), 12 out of 70 patients (17.1%, 95% CI: 8.3–26.0%) had SD and 50 out of 70 patients (71.4%, 95% CI: 60.8–82.0%) had (PD) at first radiological assessment. The overall clinical benefit rate (PR+SD) at 4 months was 20% (95% CI: 10.6–29.3). When excluding those patients who received cytotoxic chemotherapy, the response rate for first in human trials was 6.56% (95% CI: 0.35–12.77%) and the clinical benefit rate at 4 months was 11.48% (95% CI: 3.48–19.47%). Patients with TNBC derived the greatest benefit, with a response rate of 23.08% (95% CI: 6.8–39.2%) and were treated more frequently with poly (ADP-ribose) polymerase (PARP) inhibitors or chemotherapy combinations. There were no tumour responses among the 23 patients with ER-positive/HER2-negative disease.

Table 3 shows the characteristics of those patients who achieved a PR and the specific agents with evidence of anti-tumour activity. These drugs included tyrosine kinase inhibitors (TKIs) against vascular endothelial growth factor receptor (VEGFR), epidermal growth factor receptor (EGFR) pathways and PARP inhibitors alone or in combination with platinum chemotherapy. Two patients received a phase I trial as first line for metastatic disease as this comprised acceptable chemotherapy for the first-line management of MBC in combination with a targeted agent. No patients discontinued trial treatment because of toxicity and the toxic death ratio was 0%. In total, 4 patients out of 70 (5.7%) were known to have a germline mutation in BRCA2 and 1 (1.4%) was known to have a BRCA1 mutation. Overall 12 patients were treated with PARP inhibitor monotherapy. Of these, five patients with documented germline BRCA mutation had a median TTP of 5 months (range: 3–7months) (Table 4). The remaining seven patients, all with TNBC, had disease progression at first radiology assessment at 6 weeks.

**Figure 1:**
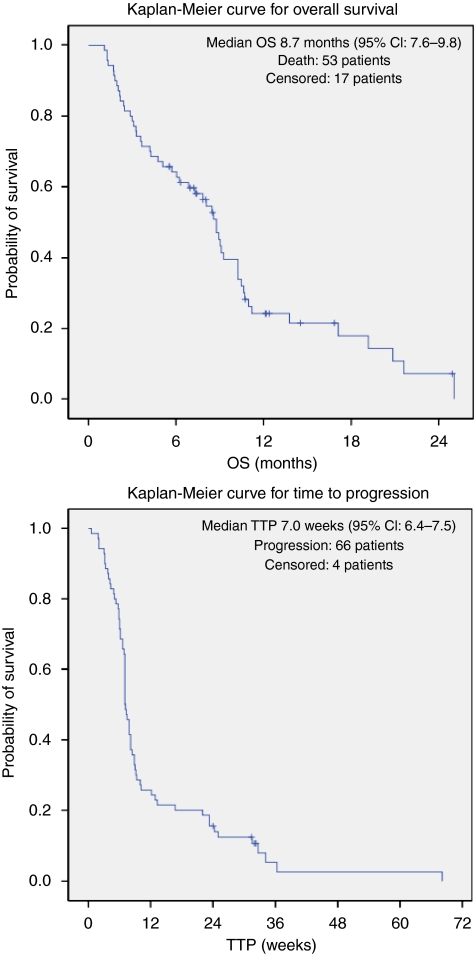
Time to progression (TTP) and overall survival (OS) Kaplan–Meier curves for the whole cohort of patients with MBC.

The median OS was 8.7 months (95% CI: 7.6–9.8 months) and the median TTP was 7.0 weeks (95% CI: 6.4–7.5 weeks) for the whole cohort of MBC patients (Figure 1). The OS univariate analysis for prognostic factors at phase I trial (Table 5) entry revealed that cumulative lines of treatment for metastatic disease, presence of liver metastases, lactate dehydrogenase (LDH) above the upper limit of normal, poor ECOG PS and low serum albumin were associated with shorter survival times. All factors remained significantly associated with a poorer prognosis in the MVA for OS. In addition, the MVA for TTP revealed that patients treated with PARP inhibitor-based trials had a significantly longer time until disease progression (Cox regression HR: 0.45 (95% CI: 0.23–0.86), *P*=0.015). Serum albumin or LDH, liver metastases, ECOG PS, heavily pretreated disease and chemotherapy-based phase 1 trials were not significantly associated with TTP in the MVA (data not shown).

## Discussion

The primary aims of phase I clinical trials in oncology are to find a dose schedule for future development of new anticancer agents and to describe the toxicity profile of these novel compounds (Roberts *et al*, 2004; Horstmann *et al*, 2005). Patients with advanced breast cancer who are offered experimental treatment at this stage usually have disease that is refractory, or has become resistant, to approved drugs and appropriate treatment recommendations remain uncertain because of the limited potential for therapeutic benefit associated with phase I trials. The survival of 8.7 months observed in this retrospective series is likely to be a reflection of the characteristics of breast cancer patients who participate in phase I trials, for example, heavily pretreated disease and multiple sites of disease at trial entry. The MVA of prognostic factors at study entry confirmed that abnormal serum LDH (a potential surrogate of disease burden), low serum albumin, poor PS, liver metastases and ⩾5 lines of previous treatment were associated with worse outcome. This supports previous retrospective and prospective reports from our institution across all tumour types (Arkenau *et al*, 2008a, 2008b, 2009). Patient selection based on prognostic factors can potentially minimise the burden to those who are the least likely to benefit from phase I treatment and therefore should not be considered for trial entry. Phase I trials frequently exclude patients with PS⩾2 and potentially other factors may be used to guide this selection.

Interestingly, the recent report from the MD Anderson Cancer Centre showed that patients with MBC referred to their Phase I unit tend to be heavily pretreated and have shorter survival times compared with a broader range of advanced cancers (Wheler *et al*, 2009). The shorter median OS of 6.7 months in comparison with our series probably results from patient heterogeneity and the intention-to-treat analysis used, which includes poor prognostic patients with complications from PD before trial entry and screening failures. Low serum albumin, heavily pretreated disease and poor PS were also confirmed to be associated with poor survival outcomes in their MBC cohort. Patient's characteristics seem to be comparable between both series, although the percentage of patients with TNBC was slightly higher in our study potentially reflecting referral of these patients for PARP inhibitor and platinum salt-based trials ongoing in our phase I unit.

Although these data from a retrospective analysis of breast cancer patients treated in phase I trials at a single institution can only be interpreted in an exploratory manner, some interesting points can be highlighted. Phase I trials have often in the past recruited a wide spectrum of patients with different tumour types. In the era of targeted therapies, however, this approach may need to change, in a number of ways. It is hoped, for example, that molecular characterisation of breast cancer will lead to more individualised treatment according to specific tumour type. In addition, 10% of patients were referred for a phase 1 trial in our analysis having only received one or two previous treatment lines for MBC, compared to 20% in the MD Anderson series. The response rate of 11.4% observed in our retrospective study combined with a significant number of patients obtaining clinical benefit, which was highest in the TNBC subtype, may suggest that referral of patients earlier in the disease course for phase I trials may be a reasonable option, particularly for patients with relatively chemo-refractory disease or subtypes of breast cancer with limited standard options.

Although the retrospective comparison should be interpreted with caution, the median TTP was significantly longer for patients treated with PARP inhibitor-based trials relative to the rest of the cohort. A total of 17 patients were treated with PARP inhibitors in this retrospective analysis. Five patients with TNBC received a PARP inhibitor in combination with a platinum salt. A majority of these patients were not heavily pretreated and thus these figures may potentially overestimate response in early phase clinical trials. In addition, 12 patients were treated with monotherapy at doses in which activity was observed in other tumour types and 5 out of these 12 patients had BRCA germline mutation and derived significant clinical benefit as shown in Table 4. Interestingly, the remaining seven patients (all with TNBC) had PD at first radiology assessment after PARP inhibitor treatment, suggesting that monotherapy alone is insufficient to offer disease control in patients without documented germline BRCA mutations. Promising activity has been confirmed with platinum salts in the treatment of TNBC (Chia *et al*, 2007a; Sirohi *et al*, 2008; Silver *et al*, 2010) and recent trials have shown PARP inhibition to be highly effective in TNBC and in patients with germline mutations of BRCA1 or BRCA2 (Fong *et al*, 2009; O’Shaughnessy *et al*, 2009).

Vascular proliferation has been shown to be an important element in tumour growth with numerous studies showing reduced survival times for patients with high levels of vascular endothelial growth factor (VEGF) expressed by breast primary tumours (Gasparini *et al*, 1997; Linderholm *et al*, 1998). Furthermore, a recent retrospective analysis showed significantly higher intra-tumoral levels of VEGF for patients with primary operable TNBC (Linderholm *et al*, 2009). In our series, there were four patients treated with TKI compounds targeted against VEGFR family members. Two patients with chemo-resistant TNBC had a radiological PR after a TKI. On the basis of these preliminary data, and recent phase II neo-adjuvant trial analysis of cisplatin and bevacizumab in TNBC (Ryan *et al*, 2009), in which a clinical CR was observed in 12 patients (26%) and a PR was observed in 24 patients (52%), prospective analysis of TKI against VEGFR in TNBC are anticipated with interest.

Mechanisms of resistance to HER2-directed therapy include impaired binding of trastuzumab to HER2, *PIK3CA* mutation and PTEN loss that promote activation of downstream receptor signalling (Stemke-Hale *et al*, 2008). One of the two patients with HER2+ trastuzumab-resistant disease in our analysis who achieved a PR received an irreversible small molecule inhibitor of EGFR and HER2. This patient was heavily pretreated with multiple lines of cytotoxics, hormonal therapy, and two lines of HER2-directed targeted therapy (trastuzumab and lapatinib). In addition, 10 patients in this series received novel agents targeting the PI3K-AKT pathway but unfortunately there were no PRs or prolonged SD to agents targeting this pathway.

This paper highlights many of the challenges in developing effective drugs for breast cancer. In particular, for this process to be as cost- and time-effective as possible, phase I trial designs need to be able to adapt to rapidly evolving changes in molecular biology and the technological advances resulting from this (Carden *et al*, 2010). These must be facilitated by validated preclinical tumour models, which are critical to aid understanding of which agents are likely to benefit in different breast cancer subtypes (Mirzoeva *et al*, 2009). In addition, integrative assessment of key oncogenic drivers at the DNA, mRNA and protein level, together with analysis of feedback loops in combination with functional genomics RNA interference approaches to elucidate drug resistance pathways (Juul *et al*, 2010) may have value in determining appropriate combinations of targeted agents and the defined tumour molecular subtypes in which to use them. These will aid the ability to conduct successful hypothesis-testing clinical trials for targeted agents in breast cancer in molecularly distinct tumour types, with better use of predictive, pharmacodynamic and response biomarkers. Early patient referral in selected tumour types and chemo-refractory disease may augment the chance of benefit to experimental therapies. In addition, selection of patients based on prognostic tools can assist go-no-go decisions on trial participation for those least likely to benefit.

## Figures and Tables

**Table 1:** Baseline characteristics

**Baseline characteristics of 70 patients**	**Total**
Median age	51.5 years (range: 34–82 years)
Median number of cycles on phase I	2 (range: 1–11 cycles)
Median lines of standard treatment	5 lines (range: 1–12 lines)
Median lines of chemotherapy	4 lines (range: 0–8 lines)
Median sites of metastatic disease	3 sites (1–5 sites)
	
*Receptor status*	
TNBC	26 (37.1%)
HER2 positive	21 (30.0%)
ER positive/HER2 negative	23 (32.9%)
	
*Previous class of chemotherapy received*	
Anthracycline	65 (92.9%)
Taxane	62 (88.6%)
Capecitabine	55 (78.6%)
Vinorelbine	38 (54.3%)
Platinum	28 (40%)
Gemcitabine	13 (18.6%)
CMF	4 (5.7%)
Other	4 (5.7%)
	
*ECOG PS at study entry*	
PS 0	22 (31.4%)
PS 1	40 (57.1%)
PS 2	8 (11.4%)

Abbreviations: ER=oestrogen receptor; HER2=epidermal growth factor receptor 2; PS=performance status; TNBC=triple-negative breast cancer.

**Table 2:** Treatment outcomes for patients based on breast cancer subtypes

	**Triple-negative breast cancer**	**ER-positive/HER2-negative breast cancer**	**HER2-positive breast cancer**	**Total/overall**
Median overall survival (95% CI)	8.5 months (4.9–12.2)	8.7 months (3.9- 13.5)	8.7 months (7.3–10.2)	8.7 months (7.6- 9.8)
Median time to progression (95% CI)	8.8 weeks (5.8–11.8)	6.5 weeks (5.7–7.3)	7.4 weeks (6.5–8.3)	7.0 weeks (6.4–7.5)
RECIST response rate	6/26 (23.0%)	0/23 (0%)	2/21(9.5%)	8/70 (11.4%)
CBR (PR+SD) at 4 months	8/26 (30.7%)	2/23 (8.7%)	4/21 (19%)	14/70 (20%)
				
*Type of phase 1 trial*				
First in human	20	22	19	61
Chemotherapy combination	6	1	2	9
				
*Target of phase 1 trial*				
DNA repair (PARP)	12	4	1	17
AKT/PI3K/ MTOR pathways	5	3	2	10
Hormone synthesis	0	8	4	12
Growth factor receptor pathways	1	0	3	4
Anti-angiogenesis	2	0	3	5
Cell cycle and apoptosis	3	0	1	4
Chromatin remodelling and antisense	0	5	1	6
Protein turnover (HSP90)	0	2	3	5
Other signalling pathways (Ras, SRC, IGF, c-met)	3	1	3	7
				
Total	26	23	21	70

Abbreviations: CBR=clinical benefit rate; CI=confidence interval; ER=oestrogen receptor; HER2=epidermal growth factor receptor 2; IGF=insulin-like growth factor; PARP=poly (ADP-ribose) polymerase; PD=progressive disease; PR=partial response; RECIST=response evaluation criteria in solid tumours; SD=stable disease; SRC=v-src sarcoma (Schmidt-Ruppin A-2) viral oncogene homolog; TNBC=triple-negative breast cancer.

**Table 3:** Characteristic of patients who responded to phase 1 treatment with single targeted agents and chemotherapy combinations

**Trial**	**Age**	**Pathology**	**Lines chem. MTX**	**Previous chem.. class received (including adj.)**	**Lines horm. MTX**	**Biological**
PARP	45	IDC, Horm–, HER−, BRCA2	1	Tax, Anthra	0	No
TKI VEGF	53	IDC, Horm–, HER−	2	Tax, Anthra, Lip. doxorubicin	0	No
TKI VEGF	58	IDC, Horm–, HER−	5	Tax, Anthra, Gem	0	No
TKI EGF/HER2	66	IDC, Horm (ER)+, HER+	7	Tax, Anthra, Cape, Plat, Gem	4	Trast/Lap
						
PARP/Carbo Paclit	48	IDC, Horm–, HER–	0	Tax, Anthra	0	No
PARP/Carbo Paclit	50	IDC, Horm–, HER–	0	Tax, Anthra	0	No
PARP/Carbo Paclit	35	IDC, Horm–, HER–	1	Tax, Anthra	0	No
SRC/Carbo Paclit	59	IDC, Horm+, HER+	5	Tax, Anthra, Cape	2	Trast

Abbreviations: adj.=adjuvant; Anthra=anthracyclines; Cape=capecitabine; Carbo=carboplatin; chem.=chemotherapy; EGF=epidermal growth factor; ER=oestrogen receptor; Gem=gemcitabine; HER2=epidermal growth factor receptor 2; horm=hormonal; IDC=invasive ductal carcinoma; Lap=lapatinib; Lip.=liposomal; MTX=metastatic disease; PARP=Poly (ADP-ribose) polymerase; Paclit=paclitaxel; Plat=platinum salts; Tax=taxanes; Trast=trastuzumab; TKI=tyrosine kinase inhibitor; TNBC=triple-negative breast cancer; VEGF=vascular endothelial growth factor.

**Table 4:** Characteristics’ of patients with germline BRCA mutation treated with PARP inhibitor monotherapy

**Patient**	**26**	**28**	**35**	**66**	**67**
TTP (months)	7	5	5	3	7^a^
Best response	SD	SD	SD	SD	PR
ER status	Negative	Positive	Negative	Positive	Negative
PR status	Negative	Negative	Negative	Negative	Negative
HER2 status	Negative	Negative	Negative	Negative	Negative
BRCA status	BRCA1	BRCA2	BRCA2	BRCA2	BRCA2
Pathology	IDC	IDC	IDC	IDC	IDC
Lines chemo MTX	4	2	1	2	1
Time MTX to phase I	22 months	16 months	20 months	27 months	12 months

Abbreviations: ER=oestrogen receptor; HER2=epidermal growth factor receptor 2; IDC=invasive ductal carcinoma; MTX=metastatic disease; PARP=Poly (ADP-ribose) polymerase; PR=progesterone receptor; TTP=time to progression.

a Ongoing PR at radiological 7-month assessment.

Partial response (PR) and stable disease (SD) according to response evaluation criteria in solid tumours (RECIST).

**Table 5:** Univariate (log-rank) and multivariate analysis (Cox regression) for OS

**Variable (*N*=70 patients, died 53)**	***N***	**Median OS (months)**	**95% CI**	**Univariate *P-*value^a^**	**MVA *P-*value^b^**	**HR (95% CI)**
Age ⩾50years	40	8.7	6.7–10.8	0.350	—	
Age< 50 years	30	8.7	7.1–10.4			
ECOG PS ⩾2	8	1.7	1.0–2.3	<0.0001	0.013	3.3 (1.2–8.6)
ECOG PS 0–1	62	8.9	8.2–9.6			
TNBC	26	8.5	4.9–12.2	0.471	—	—
HER2-positive disease	21	8.7	7.3–10.2			
ER-positive disease	23	8.7	3.9–13.5			
Previous chemo lines ⩾4	23	6.8	1.6–12.0	0.223	—	—
Previous chemo lines < 4	47	9.0	8.4–9.6			
Previous total ⩾5 lines	30	4.2	1.3–7.0	0.015	0.003	2.5 (1.3–4.8)
Previous total <5 lines	40	10.2	8.7–11.7			
Number MTX sites >2	45	8.4	7.1–9.7	0.637	—	—
Number MTX sites ⩽2	25	10.2	4.8–15.6			
Liver metastases	37	6.0	3.0–9.0	0.001	0.003	2.6 (1.3–4.9)
No liver metastases	33	10.2	7.8–12.6			
Serum albumin ⩾35 mg per 100 ml	42	10.4	8.6–12.3	<0.0001	0.002	0.35 (0.1–0.6)
Serum albumin <35 mg per 100 ml	28	3.2	2.4–4.1			
Normal serum LDH	30	10.6	7.6–13.6	<0.0001	<0.0001	3.2 (1.7–6.2)
Abnormal serum LDH	40	4.7	0.0–10.2			
Chemo-combination trial	9	7.3	0.0–19.2	0.342	—	—
First in human trial	61	8.7	7.6–9.9			
PARP inhibitor trial	17	10.6	8.4–12.8	0.055	—	—
Non-PARP trial	53	8.4	6.7–10.2			

Abbreviations: CI=confidence interval; ECOG=Eastern Cooperative Group; ER=oestrogen receptor; HER2=epidermal growth factor receptor 2; HR= hazard ratio; LDH=lactate dehydrogenase; MTX=metastatic; MVA=multivariate analysis; OS=overall survival; PARP=poly(ADP-ribose) polymerase; PS=performance status; TNBC=triple-negative breast cancer.

a Log-rank *P-*value.

b HR obtained in proportional hazards model (Cox regression) with the following terms: ECOG PS, previous chemo and treatment lines, liver metatases, albumin, LDH and PARP trial.

The table shows that ECOG PS⩾2, serum albumin <35 mg per 100 ml, presence of liver metastases, ⩾5 previous chemotherapy lines to phase 1 treatment and high serum LDH are associated with a poor OS in the univariate and multivariate analysis.
